# Robotic surgical staging for cervical cancer diagnosed during pregnancy: Immediate versus delayed definitive treatment^☆^

**DOI:** 10.1016/j.gynor.2013.03.005

**Published:** 2013-04-03

**Authors:** Christine Rojas, John W. Moroney

**Affiliations:** Wright State University, Department of Obstetrics and Gynecology, 128 Apple Street, Suite 3800 Weber CHE, Dayton, OH 45409, USA; Wright Patterson Medical Center, Department of Obstetrics and Gynecology, 4881 Sugar Maple Drive, WPAFB, OH 45433, USA

**Keywords:** Robotic assisted lymphadenectomy, Cervical cancer

## Abstract

• Definitive treatment of cervical cancer in pregnancy poses a dilemma for patients desiring to continue gestation.
• Robotic surgical staging of cervical cancer diagnosed during pregnancy is feasible.
• Robotic surgical staging improves the prognostic assessment for pregnant patients when making a decision between immediate versus delayed treatment.

## Introduction

Cervical cancer is one of the more common gynecologic malignancies diagnosed during pregnancy; the incidence in pregnancy is estimated to be approximately 1.6 to 10.6 per 10,000 pregnancies (Favero et al., 2010a). Though there are well-established treatment algorithms for the treatment of cervical cancer in non-pregnant patients, diagnosis during pregnancy poses significant challenges because of natural conflicts between the well being of the mother and fetus. Specifically, the issue of immediate vs. delayed definitive treatment can be a choice between life and death for both the mother and fetus. We present the case of a patient diagnosed with cervical cancer during the early second trimester who underwent a robotic assisted lymphadenectomy to assess for the presence of metastatic disease prior to a decision regarding immediate versus delayed definitive treatment. A review of the literature and discussion of neo-adjuvant chemotherapy as well as surgical staging for cervical cancer diagnosed during pregnancy is also provided.

## Case study

A 32 year-old Caucasian female G4P3 presented for pre-natal care at 12 + 4 weeks gestation. A speculum exam revealed a 4 cm, exophytic cervical mass. Her cervical cytology screening eighteen months prior to presentation was without evidence of dysplasia or malignancy. A bi-manual exam was notable for the absence of parametrial involvement. A cervical biopsy was consistent with poorly differentiated squamous cell carcinoma (SCC). Pelvic MRI revealed an asymmetric, 4.2 cm cervical mass without evidence of parametrial or metastatic pelvic disease; a chest x-ray was unremarkable. Obstetric ultrasound at 14 weeks gestation revealed normal fetal anatomy. Various treatment options were offered to the patient including: (1) robotic assisted trans-peritoneal lymphadenectomy prior to a decision regarding subsequent treatment, (2) Neo-adjuvant chemotherapy followed by cesarean radical hysterectomy, (3) expectant management until fetal viability followed by cesarean section and external beam radiotherapy with radio-sensitizing cisplatin (chemo-EBRT), (4) immediate chemo-EBRT or (5) radical hysterectomy with the fetus-in-situ.

**Fig. 1:**
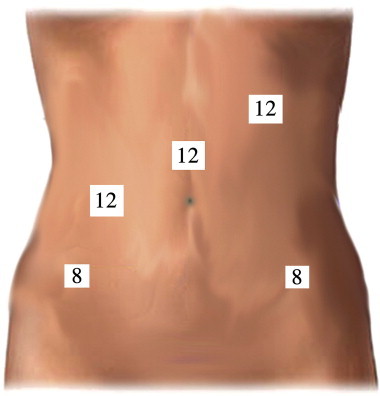
Davinci robotic system port placement and trocar size — LUQ: 5 mm trocar for insufflation and pelvic examination; 12 mm camera port 2 cm above umbilicus; 12 mm assistant port 10 cm to the right of camera port; 8 mm Davinci lateral robotic port on the left and right side of abdomen.

The patient elected to undergo a robotic assisted transperitoneal lymphadenectomy. A robotic assisted bilateral pelvic and para-aortic lymphadenectomy was performed at 15 + 1 weeks of gestation. Fig. 1 illustrates port placement of the robotic system. To facilitate access to the right and left pelvic nodal basins, the patient was secured to the operating table and placed in 40° lateral tilt prior to docking each side. Intra-operative findings were notable for a 16 week sized uterus, normal fallopian tubes, ovaries, appendix and small bowel. The resected lymph nodes were grossly normal in appearance, and her post-operative course was uncomplicated. Final pathology revealed a total of 25 lymph nodes with a single positive right external iliac lymph node. The remaining lymph nodes were negative (8 left pelvic, 12 right pelvic, and 5 para-aortic).

After extensive multi-disciplinary counseling and an institutional ethics consultation, the patient elected for immediate definitive treatment in order to maximize her survival. Planning for radiotherapy was initiated; in anticipation of this, the patient received a single dose of radio-sensitizing cisplatin. She subsequently declined radiotherapy citing emotional concerns, and was then offered a type II radical hysterectomy followed by chemo-EBRT. An uncomplicated type II radical abdominal hysterectomy through a vertical skin incision was performed; the patient was discharged to home on post-operative day #3. Final uterine pathology was notable for a 2.7 cm poorly differentiated SCC of the cervix with deep stromal invasion, extensive lymph vascular space invasion (LVSI) and un-involved margins. Three weeks post-operatively chemo-EBRT was initiated. Her radiotherapy was complicated by grade two radiation enteritis and anemia requiring transfusion. She is disease free at 18 months from the completion of therapy.

## Discussion

Cervical cancer is one of the few gynecologic malignancies for which clinical staging remains the standard of care, despite well described limitations in its ability to identify patients with asymptomatic metastatic disease. This is important because the presence of nodal disease is an extremely strong negative predictor for survival and directs therapy from surgery to chemo-EBRT. In a retrospective review of 401 women with stage 1B cervical cancer, Alvarez et al. found that 5 year survival decreased from over 90% in patients without nodal involvement to less than 70% in patients with regional lymph node involvement (Alvarez et al., 1991).

The most challenging decision for patients and physicians faced with a diagnosis of cervical cancer during pregnancy is whether or not to delay definitive treatment. Two retrospective case series (pooled n = 17) have described favorable maternal and neonatal outcomes for clinical stage 1A disease after treatment delays exceeding a mean of 18 weeks; all of these patients (17/17) were without evidence of disease after a mean follow-up of 136 months (Takushi et al., 2002; Sood et al., 1996). Based on this, it appears that even prolonged delays in definitive treatment of patients with clinical stage 1A disease diagnosed during the late first, or early second trimester of pregnancy are unlikely to negatively affect maternal mortality.

Patients diagnosed with stage II/III disease are much more likely to have either clinically evident or occult metastatic disease at presentation, and outcomes for such patients diagnosed in pregnancy are correlatively poor. Fruscio et al. described in their literature review from 1996 to 2012 the outcomes for six stage II/III patients treated with neo-adjuvant chemotherapy with a mean treatment delay of 14 weeks (Table 1) (Fruscio et al., 2012). Over a mean follow-up of 28 (range 5–80) months, 3 of 6 were dead of disease and 1 was alive with disease, representing a 66% treatment failure rate (Table 1) (Fruscio et al., 2012). Conversely, Takushi described a series of 6 patients with stage II/III disease who were treated definitively at presentation (mean gestational age 17.5 weeks, range 6–37) (Takushi et al., 2002). Five of six of these patients were without evidence of disease at a mean follow-up of 86 (range 4–120) months (Takushi et al., 2002). Based on these series it would be appropriate to counsel patients that there is a significant survival advantage favoring immediate treatment in patients diagnosed with stage II/III disease during pregnancy.

The prognosis for stage 1A and stage II/III in patients diagnosed with cervical cancer during pregnancy, both with immediate and delayed treatment, is simpler to predict than for patients with stage 1B disease. This is likely related to the fact that stages 1B1 and 1B2 patients have significantly different node positivity rates. Among a pooled series of patients with stage 1B cancers treated with neo-adjuvant chemotherapy followed by cesarean delivery and definitive therapy (n = 26) (Table 2), 1/12 (8%) of 1B1 patients and 4/14 (28%) of 1B2 patients were found to have positive nodes at time of cesarean hysterectomy + lymphadenectomy (Fruscio et al., 2012). Among these 26 patients, one refused hysterectomy and lymphadenectomy after delivery. Also, the nodal status was not available for five of the 26 patients in this series. Similarly, significantly disparate outcomes after neo-adjuvant chemotherapy were appreciable between stages 1B1 and 1B2 patients. In the same pooled series, 1/12 (8%) of stage 1B1 patients and 5/14 (35%) of stage 1B2 patients were dead of disease (DOD) at a mean follow-up of 40 (27–59) months (Fruscio et al., 2012).

Because nodal status plays such a pivotal role in determining prognosis, particularly when considering a delay in definitive treatment, several authors have described antenatal surgical staging prior to initiating either expectant management or neo-adjuvant chemotherapy until fetal viability. Outcomes following antenatal surgical staging have been described for twenty-eight patients (Favero et al., 2010a, 2010b; Alouini et al., 2008). Among women (mean age = 32) with stage 1B disease (n = 23), 3/19 (16%) of stage 1B1 patients and 2/4 (50%) of stage 1B2 patients were found to have positive nodes (Favero et al., 2010a, 2010b; Alouini et al., 2008). Two out of five (40%) node positive patients, irrespective of clinical stage at diagnosis, were DOD at a mean follow-up of 26 (range 17–36) months (Alouini et al., 2008; Favero et al., 2010b). One of the five patients with positive nodes (clinical stage 1B1 disease) was lost to follow-up (Alouini et al., 2008; Favero et al., 2010b). Based on this data, antenatal surgical staging data does provide an opportunity to refine a patient's prognosis and offer tailored guidance for those patients who are considering immediate vs. delayed treatment.

This is the first published description of robotic assisted transperitoneal lymphadenectomy for surgical staging of cervical cancer during pregnancy. In this case, prognostic data provided by surgical staging refined prognostic counseling for this patient, informing her difficult decision regarding immediate vs. delayed definitive treatment in the context of a desired pregnancy. While a broader discussion of clinical vs. surgical staging for cervical cancer diagnosed during pregnancy is beyond the scope of this discussion, this case, and previous series describing laparoscopic surgical staging in pregnant patients have shown that it is feasible, safe and provides significant prognostic data to inform patient decisions about immediate vs. delayed treatment for stage 1B cancers. With robotic surgery becoming an increasingly utilized tool for minimally invasive surgery, this case serves to validate its use in pregnant patients requiring advanced laparoscopic techniques.

## Conflict of interest statement

The authors declare that there are no conflicts of interest.

## Consent

A written informed consent was obtained from the patient for publication of this case report.

## Figures and Tables

**Table 1:** Reported cases of neoadjuvant therapy followed by cesarean-radical hysterectomy for cervical cancer stages II–III during pregnancy: literature review (1996–2012).

Author	Year	# of cases	Clinical stage at Dx	Gestational age at Dx (weeks)	Treatment delay (weeks)	LN status at delivery	Neoadjuvant chemo	Treatment after pregnancy	Mother's outcome
Tewari	1998	1	IIA	16	18	Negative	Cis/vinc	RH/RT	DOD
Marana	2001	1	IIB	14	24	N/A	Cis/bleo	None^a^	DOD
Palaia	2007	1	IIB	19	16	Negative	Cis	RH	NED
Bader	2007	1	IIA	19	14	Positive	Cis/vinc	RH/CT	NED
Benhaim	2008	1	IIIB	22	6	N/A	Cis	RH/CT/RT	DOD
Chun	2010	1	IIA	28	5	Negative	Carb/pac	RH	AD

LN status was determined after pregnancy; Dx — diagnosis. RT — radiotherapy; Cis — cisplatin; Pac — paclitaxel; carb — carboplatin; Vinc — vincristine; bleo — bleomycin; RH — radical hysterectomy; CT — chemotherapy; NED — no evidence of disease; DOD — death of disease; AD — alive with disease; LN — lymph node; N/A — not available.

a Patient refused radical hysterectomy, lymphadenectomy and radiation treatment.

**Table 2:** Reported cases of neoadjuvant therapy followed by cesarean-radical hysterectomy for cervical cancer stages 1B1 and 1B2 during pregnancy: literature review (1996–2012).

Author	Year	# of cases	Clinical stage at Dx	Gestational age at Dx (weeks)	Treatment delay (weeks)	LN status at delivery	Neoadjuvant chemo	Treatment after pregnancy	Mother's outcome
Giacalone	1996	1	1B1	17	15	Negative	Cis	RH	NED
Lai	1997	2	1B2	N/A	N/A	Positive	Cis/vinc/bleo	RH	DOD
			1B2	12	N/A	Negative	Cis/vinc/bleo	RH	DOD
Tewari	1998	1	1B2	21	11	Negative	Cis/vinc	RH	NED
Caluwaerts	2006	1	1B1	15	17	Negative	Cis	RH	NED
Karam	2007	1	1B2	23	10	Negative	Cis	RH/CT/RT	NED
Rabaiotti	2010	1	1B2	15	17	Positive	Cis	RH/CT/RT	DOD
Favero	2010	5	1B1	14	19	–	Cis	N/A	NED
			1B1	18	14	–	Cis	N/A	NED
			1B1	22	14	–	Cis	N/A	NED
			1B1	14	18	–	Cis	N/A	N/A
			1B1	18	16	–	Cis	N/A	N/A
Chun	2010	2	1B1	25	10	Negative	Cis/pac	RH	DOD
			1B2	28	8	Positive	Cis/pac	RH/CT	NED
Smyth	2010	1	1B2	23	12	N/A	Adria/CTX	N/A^a^	NED
Li	2011	2	1B2	27	6	Negative	Cis/pac	RH/CT/RT	NED
			1B2	29	5	Negative	Cis/pac	RH	NED
Fruscio	2012	9	1B1	22	14	Negative	Cis	RH	NED
			1B1	20	16	Negative	Cis	RH	NED
			1B1	26	10	Negative	Cis	RH	NED
			1B1	8	28	Positive	Cis	RH/CT/RT	NED
			1B2	20	15	Negative	Cis	RH/CT/RT	DOD
			1B2	16	18	Negative	Cis/Pac	RH	NED
			1B2	16	17	Negative	Cis/Pac	RH	NED
			1B2	18	14	Negative	Cis	RH	NED
			1B2	13	17	Positive	Cis/vinc	RH/RT	DOD

LN status was determined after pregnancy; Dx — diagnosis. RT- radiotherapy; Cis — cisplatin; Pac — paclitaxel; carb — carboplatin; Vinc — vincristine; bleo — bleomycin; adria — adriamycin; CTX — cyclophosphamide; RH — radical hysterectomy; CT — chemotherapy; NED — no evidence of disease; DOD — death of disease; AD — alive with disease; LN — lymph node; N/A — not available.

a Radical hysterectomy and lymphadenectomy were not performed.
